# Clinical trajectories of hand function impairment in systemic sclerosis: an unmet clinical need across disease subsets

**DOI:** 10.1136/rmdopen-2023-003216

**Published:** 2024-01-12

**Authors:** Enrico De Lorenzis, Vishal Kakkar, Stefano Di Donato, Michelle Wilson, Theresa Barnes, Chris Denton, Emma Derrett-Smith, Karen Douglas, Philip Helliwell, Ariane L Herrick, Benazir Saleem, Muhammad Nisar, Catherine Morley, Lorraine Green, Begonya Alcacer-Pitarch, Francesco Del Galdo

**Affiliations:** 1 Leeds Institute of Rheumatic and Musculoskeletal Medicine, University of Leeds, Leeds, UK; 2 Division of Rheumatology, Catholic University of the Sacred Heart, Fondazione Policlinico Universitario A. Gemelli, Rome, Italy; 3 NIHR Leeds Biomedical Research Centre, Leeds Teaching Hospitals NHS Trust, Leeds, UK; 4 Department of Rheumatology, Countess of Chester Hospital NHS Foundation Trust, Liverpool, UK; 5 Department of Rheumatology, Royal Free London NHS Foundation Trust, London, UK; 6 Department of Rheumatology, University Hospitals Birmingham NHS Foundation Trust, Birmingham, UK; 7 Department of Rheumatology, The Dudley Group NHS Foundation Trust, Dudley, UK; 8 Department of Rheumatology, St. Luke’s Hospital, Bradford Teaching Hospitals NHS Foundation Trust, Bradford, UK; 9 Centre for Musculoskeletal Research, The University of Manchester, Salford Royal NHS Foundation Trust,Manchester Academic Health Science Centre, Manchester, UK; 10 Department of Rheumatology, York Teaching Hospital NHS Foundation Trust, York, UK; 11 Department of Rheumatology, Luton & Dunstable University Hospital NHS Foundation Trust, Luton, UK; 12 Department of Rheumatology, South Tyneside and Sunderland NHS Foundation Trust, Sunderland, UK

**Keywords:** Systemic Sclerosis, Outcome Assessment, Health Care, Patient Reported Outcome Measures

## Abstract

**Background:**

Hand involvement is an early manifestation of systemic sclerosis (SSc), culprit of diagnosis and classification, and recognised major driver of disability. Impairment of hand function burdens both limited and diffuse cutaneous subsets and therefore could be targeted as ‘basket’ endpoint in SSc. Nevertheless, its natural history in current standard of care is not well characterised, limiting the design of targeted trials. The aim of this study is to describe prevalence, natural history and clinical factors associated with hand function deterioration in a longitudinal, multicentre, observational SSc cohort.

**Methods:**

Hand function was captured through the validated Cochin Hand Function Scale in patients consecutively enrolled in a multicentre observational study and observed over 24 months. Minimal clinically important differences and patient acceptable symptom state were analysed as previously described.

**Results:**

Three hundred and ninety-six consecutive patients were enrolled from 10 centres; 201 with complete follow-up data were included in the analysis. Median (IQR) disease duration was 5 (2–11) years. One hundred and five (52.2%) patients reported clinically significant worsening. Accordingly, the proportion of patients reporting unacceptable hand function increased over 2 years from 27.8% to 35.8% (p<0.001). Least absolute shrinkage and selection operator analysis identified male gender, disease subset, Raynaud’s Condition Score, tenosynovitis and pain, as some of the key factors associated with worsening hand involvement.

**Conclusions:**

Hand function deteriorates over time in more than 50% of SSc patients despite available therapies. The analysis of factors associated with hand function worsening supports the involvement of both inflammation, vascular and fibrotic processes in hand involvement, making it a hallmark clinical manifestation of SSc. Our data are poised to inform the design of intervention studies to target this major driver of disability in SSc.

**WHAT IS ALREADY KNOWN ON THIS TOPIC**

- Hand is a major target organ and driver of disability in both limited and diffuse cutaneous systemic sclerosis (SSc).
- Hand impairment is known to be multifactorial in SSc but its natural history in the current standard of care is not well characterised.

**WHAT THIS STUDY ADDS**

- Hand function deterioration rate over 2 years and its clinical predictability were defined. Hand disability progression despite current standard of care emerged as unmet clinical need in SSc patients.

**HOW THIS STUDY MIGHT AFFECT RESEARCH, PRACTICE OR POLICY**

- Our data are poised for patient risk stratification in clinical practice and to inform the design of intervention studies to target this hand disability in SSc.

## Introduction

Functional hand impairment is a primary determinant of disability in systemic sclerosis (SSc) patients^1 2^ relevant to both limited and diffuse cutaneous clinical subsets of the disease. Reduced hand function starts very early as the consequence of Raynaud’s phenomenon (RP) and later in the natural history due to severe inflammation or fibrosis of skin,^3^ digital ulcers,^4^ calcinosis,^5 6^ and inflammation or fibrosis of joints and periarticular tissues.^7 8^

Despite this profound impact on the disease and the reported clinical association in cross-sectional populations, the prevalence and rate of deterioration in hand function over time in SSc patients is largely unknown, as well as the clinical and demographic factors and treatments associated with its worsening over time. Furthermore, the effects of immunosuppressive and vasoactive treatment on SSc hand function are also poorly determined.

The Cochin Hand Function Scale (CHFS) is among the most accepted tools for hand disability assessment, and it has been extensively adopted in SSc disease.^9–11^ This tool is based on patient-reported disability and includes comprehensive questions validated to summarise the level of difficulty in performing specific daily tasks.

The objective of this study is to describe hand function changes according to CHFS over a 24-month interval, observed in a real-life cohort of SSc patients treated according to current standard of care. A further objective was to identify the baseline clinical and demographic factors that are associated with poor hand function as well as its deterioration over 24 months.

## Methods

### Study design and patients

This study is a predefined analysis of a longitudinal observational cohort. The Transparent Reporting of a multivariable prediction model for Individual Prognosis Or Diagnosis checklist was used to define the study design.^12^ Patients were not directly involved in the design of the study, but the unmet clinical needs that emerged during routine clinical practice were taken into account. Patients were informed of the results of this study on request.

Consecutive patients enrolled in Stratification for Risk of Progression in systemic sclerosis between January 2016 and December 2018 were included in the final analysis if they (i) fulfilled the ACR/EULAR classification criteria for SSc,^13^ (ii) had available a baseline and a longitudinal evaluation after 24±3 months and (iii) did not have an end-stage hand disability defined for previous digital amputations or plastic/orthopaedic hand surgery.

Within the comprehensive medical assessment as per the EUSTAR Minimal Essential Dataset^14^ specific variables were analysed as putative predictors according to previous literature and clinical plausibility.

### Physician assessment of hand involvement

Hand physical examination parameters analysed included skin thickness, digital ulcers, calcinosis, tenosynovitis and flexion contractures.

Skin thickness of the hand was assessed as modified Rodnan Skin Scores (mRSS) values of fingers, hands and forearms that we defined as distal skin score (range 0–18). The minimal clinically significant increase of mRSS in patients affected by the diffuse cutaneous variant of SSc was defined as a 5-point absolute increase in patients with a baseline mRSS<20, and a relative 25% increase in the other patients.^15^

Digital ulcers were reported as loss of skin continuity involving at least the epidermis and basal membrane of any area of skin covering the digits, including those overlying calcinotic lesions and bony prominences.^16^ Calcinosis was reported when revealed by routine clinical examination, following surgical curettage or hand X-ray. Hand tenosynovitis was reported as the presence of tender and swollen radiocarpal, metacarpal, or interphalangeal joints or hand tendons.^17^ Flexion contractures were reported in the presence of fixed flexion of proximal and or distal interphalangeal joints on at least one finger. History of digital ulcer, tenosynovitis and calcinosis was also analysed.

Capillaroscopy data were acquired through VideoCap V.3.0 capillaroscope (DS Medica, Milan, Italy) and pattern was classified as non-specific, early, active or late as previously described.^18^

### Additional clinical information

Pulmonary function tests (PFTs) were also collected and reported as part of routine clinical assessment in SSc. The functional deterioration of lung function over 12-month intervals in patients with evidence of interstitial lung disease (ILD) on the high-resolution CT was defined according to OMERACT criteria as a relative reduction of the forced vital capacity (FVC) by 10%, or a 5% reduction in combination with a relative reduction of the alveolar diffusion capacity of carbon monoxide (DLco) by 15%.^19^

Clinical information about the 5-year SSc-related mortality was also collected as additional exploratory outcome measure. The cause of death was determined based on the available digital clinical files for all enrolled patients. Each episode of decease was classified as either an SSc-related death or a death related to an alternative cause. SSc-related deaths included scleroderma renal crisis, pulmonary superinfection, right-sided heart failure, end-stage respiratory failure, acute ischaemic event or major arrhythmia in patients with severe ILD, RHC-proven pulmonary arterial hypertension or CMRI-defined primary SSc heart involvement at the last available evaluation. Severe SSc-ILD was defined as FVC≤80% and DLco≤50% at the most recent evaluation.^20^ Deaths due to cancer, infective complications or cardiovascular events in patients without severe cardiopulmonary involvement, as well as deaths classified as senectus (death at home in patients over 80 without any of the previous conditions), were not considered SSc-related.

### Patient-reported outcomes

The CHFS was collected and analysed as previously described.^21^ Availability of CHFS assessment at baseline and 24±3 months) was the only criteria for identification of patients to be analysed within the cohort. When 12±3-month evaluation was missing, it was imputed from baseline and 24±3-month evaluations.

CHFS thresholds for patient acceptable symptom state (PASS) as well as minimal clinically important difference (MCID) for worsening (MCID-Worsening) and improvement (MCID-Improvement) were identified as previously described.^22^ Specifically, the 26-point cut-off of total CHFS was employed as PASS. The MCID-Worsening was defined in case of simultaneous 21.6% relative and a 1.4-point absolute increase in CHFS whereas MCID- Improvement, for a simultaneous 13.1% relative and a 3.4-point absolute decrease in CHFS. Only the absolute changes were considered for patients presenting a baseline CHFS=0.

Baseline global disability according to Health Assessment Questionnaire disability index (HAQ-DI),^23^ pain intensity on 100 mm visual analogue scale (VAS) and Raynaud’s Condition Score (RCS) were also analysed. A VAS score ≥75 mm was adopted to define high-intensity pain, and RCS threshold of 49 points was used to define severe RP as previously published.^24 25^

### Standard of care treatment

All enrolled patients were treated according to international recommendations^26^ and National Health System standard of care. The participation in the study did not directly or indirectly affect any treatment choices. Medication exposure at baseline was categorised in vasoactive treatments (calcium channel blockers, endothelin receptor antagonists, phosphodiesterase-5 inhibitors and iloprost), immunosuppressants (cyclophosphamide, mycophenolate mofetil, azathioprine, methotrexate, rituximab and tocilizumab), and second-line analgesic treatments according to WHO classification,^27^ namely weak oral opioids (eg, tramadol or codeine), α2δ subunit calcium channel blockers (eg, pregabalin or gabapentin), or antidepressants (eg, duloxetine or amitriptyline). For the purposes of this evaluation, the patients were considered as exposed to a drug after at least 3 months of oral treatment, and one cycle of at least two 375 mg/m^2^ infusions of or a cumulative dose of at least 1 g of cyclophosphamide in the previous 6 months.

All subjects with skin ulcers underwent local wound debridement and dressing according to standardised approach of Connective Tissue Disease Digital Ulcer Clinic of Leeds Teaching Hospital Trusts. None of the patients participated in a regular, repeated hand rehabilitation programme.

### Statistical analysis

Categorical variables were reported as numbers and percentages. Continuous variables as mean±SD for normal distributed data or median and IQR for skewed data, according to the distribution of the data assessed by inspection of quantile–quantile (Q–Q) plots. Predictive mean matching was used as imputation method for missed CHFS observations at the 12-month timepoint based on values observed at baseline and 24-month timepoints and generation of five imputed datasets.

Given the aim of the study, CHFS changes over 24 months and cumulative incidence of MCID-Worsening over 24 months (ie, patients meeting MCID-Worsening threshold in at least one of the two consecutive 12-month intervals) were chosen as outcome measures.

The differences in continuous variables among distinct clinical groups at baseline were investigated using Student’s t-test or Mann-Whitney U test for independent groups according to the data distribution and the homogeneity of variances assessed by Levene’s test. Pearson’s or Spearman’s (Rs) correlation coefficients were adopted to explore the relationship between CHFS and continue variables as appropriate, based on data distribution and presence of outliers. The comparison of categorical variables was performed with the Pearson χ^2^ test or Fisher’s exact test, according to the total number of observations for each combination of variables.

The comparison of the proportion of patients with CHFS≥PASS and CHFS values over the three timepoints was performed by McNemar’s χ^2^ and one-way repeated measure analysis of variance (ANOVA), respectively. Since the assumptions of outlier absence, normal distribution, homogeneity of variance and sphericity according to Mauchly’s test were not extensively fulfilled, the one-way repeated measures ANOVA was conducted on 10% trimmed means. A two-way mixed ANOVA on 10% trimmed means was performed to determine whether CHFS changes at the three timepoints differed in groups of patients identified by the main clinical variables.^28^ Differences in CHFS distribution at the baseline, 12-month and 24-month timepoints were visualised through density plot. Median CHFS values with corresponding IQRs for alternative disease characteristics were reported at the antipodes of a Kiviat chart for baseline and 24-month timepoints for a visual comparison.

Alluvial plots were used for the descriptive analysis to visualise hand function trajectories through changes in frequency distributions of CHFS≥PASS as well as MCID-Worsening and MCID-Improvement over time. The least absolute shrinkage and selection operator (LASSO) model^29^ was used to select the baseline clinical variables associated with the occurrence of MCID-Worsening in any of the two consecutive 12-month intervals. All the clinical information available at baseline were evaluated as predictors and continuous measures were preliminary standardised. A preliminary manual sorting of variables to be evaluated for the model was performed to select continuous over the corresponding categorical variables to avoid information loss, and to prefer measures of contingent clinical involvement over those related to previous unactive clinical manifestations (eg, current over any history of digital ulcers). The linear relationship between the logit of the outcome and each continuous predictor variable was verified by inspection of the corresponding scatterplot. The absence of influential observation was confirmed in the absence of standardised residual ≥3 SD and multicollinearity of predictors in the absence variance inflation factor values ≥5. The minimal penalty term λ of the LASSO model was selected using a 10-fold cross validation and by the least mean square error in the cohort.

A 5-year competing risk survival analysis was also explored to investigate SSc-related death from alternative causes of death. The cumulative incidence function was applied to account for the competing event, with deaths due to other causes considered as competing risks for SSc-related mortality. The cumulative incidence of the primary endpoint was quantitatively compared between patients who experienced or did not experience CHFS MCID-Worsening using Fine-Gray competing risk regression with sub-HR (sHR) and 95% CIs as summary statistics.^30^

### Sample size considerations

The sample size calculation was made on a repeated measure ANOVA with two within-subjects factors (two groups for clinical variable and three timepoints), a power of 90%, and an α of 0.05. The non-sphericity correction coefficient was set at 0.55 and the correlation coefficient among repeated measures was assumed at 0.5. Further, in the absence of published data on the interaction between clinical variables and longitudinal CHFS changes in SSc and given the adjustment provided by trimming, the effect size for interaction effect was postulated to be small (0.142, partialη^2^=0.02).

The sample size was estimated to be 162. Given the use of 10% trimmed means, the 162 minimal sample size should be inflated to 194, hence our sample of 201 was deemed appropriate for the analysis. A sample size of 201 was considered adequate to build a prediction model with up to 20 potential predictors and a 50% cumulative incidence of MCID-Worsening (100 expected observed events).^31^

Statistical significance was defined as a p<0.05 for all the statistical analyses and all the tests were two-tailed. The sample size calculations were performed with G*Power V.3.1.9.6 while the statistical analysis with RStudio V.2022.02.3.

## Results

### Study cohort clinical features and association with hand disability

**Figure 1:**
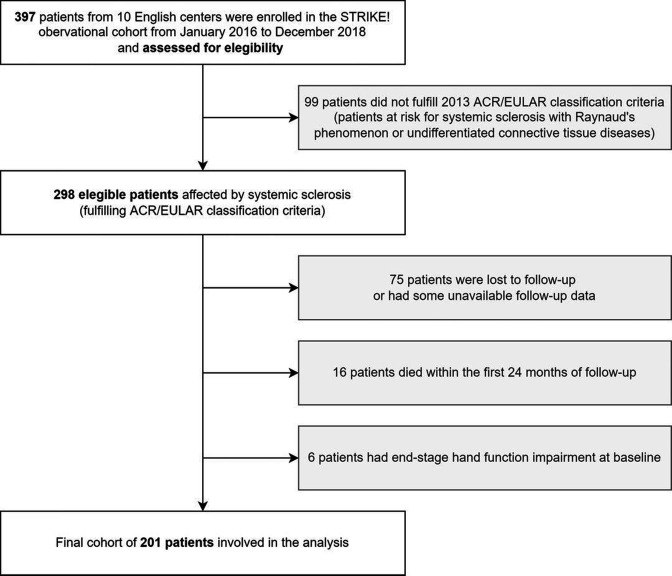
Patient selection within STRIKE! cohort according to the defined enrolment criteria. STRIKE, Stratification for Risk of Progression in systemic sclerosis.

The flowchart of patient selection and the characteristics of the 201 patients included in the final analysis are reported in figure 1 and table 1, respectively. Baseline CHFS had a non-normal distribution (online supplemental figure 1). Median (IQR) baseline CHFS of the study population was 12 (IQR 2.0–29.0). Fifty-six patients (27.9%) reported an unacceptable baseline severity of hand symptoms severity according to CHFS≥PASS. CHFS≥PASS status at baseline was associated in univariate analysis with the diffuse cutaneous variant, a more severe capillaroscopy impairment, a history of prior or current digital ulcers, a more severe RP, pain, global disability according to HAQ-DI, and exposure to second-line medications for chronic pain control. Moreover, patients with a CHFS≥PASS status presented lower FVC, total lung capacity and DLco compared with the rest of the patient sample (table 1).

10.1136/rmdopen-2023-003216.supp1 Supplementary data

**Table 1:** Baseline characteristics of the enrolled patients

Baseline hand disability				
	Overall N=201	Acceptable (CHFS<PASS) N=145	Unacceptable (CHFS≥PASS) N=56	P value
Age, years, mean±SD	55.7±12.2	55.4±12.3	56.5±12.0	0.6
Gender				0.1
Female	174 (86.6%)	129 (89.0%)	45 (80.4%)	
Male	27 (13.4%)	16 (11.0%)	11 (19.6%)	
Le Roy variant				**0.012**
Diffuse cutaneous	60 (29.9%)	36 (24.8%)	24 (42.9%)	
Limited cutaneous	141 (70.1%)	109 (75.2%)	32 (57.1%)	
Disease duration, years, median (IQR)	5.0 (2.0–11.0)	5.0 (2.0–10.0)	6 (2–11)	0.5
ACA positive	76 (37.8%)	56 (38.6%)	20 (35.7%)	0.7
Anti-Scl70 antibody positive	90 (44.8%)	71 (49.0%)	19 (33.9%)	0.055
Early, active or late SSc capillaroscopy pattern	182 (90.5%)	129 (89.0%)	53 (94.6%)	0.2
Active or late capillaroscopy pattern	161 (80.1%)	110 (75.9%)	51 (91.1%)	**0.015**
Late capillaroscopy pattern	88 (43.8%)	53 (36.6%)	35 (62.5%)	**<0.001**
Current digital ulcers	28 (13.9%)	16 (11.0%)	12 (21.4%)	0.056
Positive history of digital ulcers	98 (48.8%)	60 (41.4%)	38 (67.9%)	**<0.001**
Current hand calcinosis	28 (13.9%)	20 (13.8%)	8 (14.3%)	0.9
Positive history of hand calcinosis	76 (37.8%)	52 (35.9%)	24 (42.9%)	0.4
Current hand tenosynovitis	17 (8.5%)	9 (6.2%)	8 (14.3%)	0.088
Positive history of hand tenosynovitis	22 (10.9%)	13 (9.0%)	9 (16.1%)	0.2
Current flexion contractures	22 (10.9%)	12 (8.3%)	10 (17.9%)	0.051
mRSS, median (IQR)	2.0 (0.0–5.0)	2.0 (0.0–5.0)	4.0 (2.0–8.0)	**0.006**
Skin score up to elbows, median (IQR)	2.0 (0.0–4.0)	2.0 (0.0–4.0)	3.0 (2.0–6.0)	**<0.001**
VAS pain, mm, median (IQR)	40 (10–60)	20 (5–50)	60 (40–70)	**<0.001**
VAS pain≥75 mm	24 (11.9%)	11 (7.6%)	13 (23.2%)	**0.002**
RCS, median (IQR)	40 (10–60)	30 (10–50)	60 (28–80)	**<0.001**
RCS≥49	90 (44.8%)	53 (36.6%)	37 (66.1%)	**<0.001**
HAQ-DI, median (IQR)	1.125 (0.375–1.875)	0.875 (0.250–1.250)	2.125 (1.725–2.525)	**<0.001**
ILD on HRCT	66 (32.8%)	42 (29.0%)	24 (42.9%)	**0.060**
FVC, %, mean±SD	103.6±22.6	106.9±21.0	95.0±24.3	**0.002**
TLC, %, mean±SD	94.0±17.3	96.2±16.0	88.1±19.3	**0.007**
DLco, %, mean±SD	62.0±15.6	64.5±15.4	55.2±13.9	**<0.001**
Kco, %, mean±SD	80.3±15.3	80.8±14.2	79.3±17.9	0.7
Immunosuppressive treatment	119 (59.2%)	80 (55.2%)	39 (69.6%)	0.061
Vasoactive treatment	161 (80.1%)	112 (77.2%)	49 (87.5%)	0.1
Second-line analgesic treatment	85 (42.3%)	46 (31.7%)	39 (69.6%)	**<0.001**
Baseline CHFS, median (IQR)	12 (2.0–29.0)	4.0 (1.0–13.0)	41.0 (36.0–48.2)	**<0.001**

Bold values denote statistical significance at the p < 0.05 level.

ACA, anti-centromere antibody; CHFS, Cochin Hand Function Scale; DLco, alveolar diffusion of carbon monoxide; FVC, forced vital capacity; HAQ-DI, Health Assessment Questionnaire disability index; HRCT, high-resolution CT; ILD, interstitial lung disease; Kco, carbon monoxide transfer coefficient; mRSS, modified Rodnan Skin Score; PASS, patient acceptable symptom state; RCS, Raynaud’s Condition Score; TLC, total lung capacity; VAS, visual analogue scale.

Beyond PASS cut-off, continuous higher baseline CHFS values were reported in the subgroups of patients presenting diffuse cutaneous variant, ACA negativity, late SSc capillaroscopy pattern, positive history of digital ulcers, current tenosynovitis and flexion contractures, VAS pain ≥75 mm, RCS≥49 and baseline exposure to immunosuppressant, vasoactive and second-line analgesic treatments (table 2). At baseline, CHFS values showed also a statistically significant correlation with HAQ-DI (Rs=0.739, p<0.001), VAS pain (Rs=0.535, p<0.001), RCS (Rs=0.447, p<0.001), distal skin score (Rs=0.269, p<0.001) but not with disease duration or age.

**Table 2:** CHFS differences according to baseline clinical subsets

		Timepoints			P value	
		Baseline CHFS median (IQR)	12-month CHFS median (IQR)	24-month CHFS median (IQR)	Baseline comparison	Timepoint interaction
Gender	Male	17.0 (2.0–38.0)	24.0 (9.5–38.0)	25.0 (5.0–38.0)	0.3	0.6
	Female	10.5 (2.0–27.5)	14.0 (2.0–29.0)	15.0 (3.0–31.8)		
LeRoy cutaneous variant	Diffuse	20.5 (3.0–41.0)	24.5 (10.0–41.2)	28.0 (6.8–39.5)	**0.002**	0.7
	Limited	7.0 (1.0–23.0)	10.0 (2.0–27.0)	12.0 (2.0–28.0)		
Anti-Scl70 antibody	Positive	14.0 (2.0–39.0)	22.0 (3.0–36.0)	19.0 (3.0–36.0)	0.18	0.9
	Negative	11.0 (1.0–25.2)	14.0 (2.0–27.0)	15.0 (2.0–32.0)		
ACA	Positive	6.0 (1.0–22.8)	10.5 (1.0–27.0)	12.0 (2.0–29.0)	**0.039**	0.9
	Negative	14.0 (2.0–36.0)	19.0 (4.0–32.5)	19.0 (3.0–37.0)		
Capillaroscopy pattern	Non-late	5.0 (1.0–21.0)	11.0 (2.0–25.0)	12.0 (2.0–28.0)	**<0.001**	0.072
	Late	20.0 (3.0–39.0)	23.0 (4.0–40.5)	21.0 (3.0–39.0)		
Current digital ulcers	Yes	24.0 (4.8–37.0)	25.0 (4.5–42.5)	24.5 (5.5–39.0)	**0.014**	**0.046**
	No	9.0 (1.0–26.0)	14.0 (2.0–29.2)	14.0 (2.0–31.0		
Any history of digital ulcers	Yes	20.5 (3.0–40.5)	23.0 (6.2–37.5)	22.5 (6.0–39.0)	**<0.001**	0.7
	No	6.0 (0.5–19.0)	9.0 (1.0–24.0)	8.0 (1.0–25.0)		
Current hand tenosynovitis	Yes	24.0 (21.0–37.0)	25.0 (11.0–32.0)	29.0 (16.0–37.0)	**0.002**	0.099
	No	9.0 (1.0–26.5)	14.5 (2.0–29.2)	14.0 (2.0–31.5)		
Any history of hand tenosynovitis	Yes	23.0 (13.5–36.8)	23.0 (9.5–31.0)	24.0 (9.0–37.0)	**0.016**	0.125
	No	9.0 (1.0–27.0)	15.0 (2.0–29.5)	14.0 (2.0–31.5)		
Current hand calcinosis	Yes	16.0 (2.0–27.2)	18.5 (3.8–27.5)	18.5 (3.8–32.2)	0.4	0.7
	No	11.0 (2.0–29.0)	14.0 (2.0–30.0)	16.0 (2.0–32.0)		
Any history of hand calcinosis	Yes	14.5 (2.8–31.0)	19.0 (4.8–32.2)	18.5 (5.0–36.2)	0.085	1.0
	No	7.0 (1.0–26.0)	13.0 (2.0–28.0)	14.0 (2.0–31.0)		
Current flexion contractures	Yes	22.5 (7.0–44.0)	27.0 (7.2–45.0)	30.5 (6.2–51.2)	**0.014**	0.5
	No	9.0 (1.0–26.0)	14.0 (2.0–28.5)	14.0 (2.5–30.5)		
VAS pain	≥75 mm	28.5 (6.3–45.0)	26.5 (21.0–46.2)	37.0 (23.2–50.0)	**0.001**	0.3
	<75 mm	9.0 (1.0–25.0)	13.0 (2.0–28.0)	13.0 (2.0–29.0)		
RCS	≥49	21.5 (7.0–41.0)	26.5 (13.0–41.5)	28.0 (16.2–42.5)	**<0.001**	0.181
	<49	4.0 (0.0–18.5)	5.0 (0.5–20.5)	5.0 (0.0–21.0)		
Immunosuppressive treatment	Yes	15.0 (3.0–36.0)	21.0 (5.0–32.5)	22.0 (6.0–37.0)	**0.004**	**0.034**
	No	4.5 (0.0–22.0)	9.0 (1.0–26.0)	5.5 (1.0–24.5)		
Vasoactive treatment	Yes	13.0 (2.0–35.0)	19.0 (3.0–31.0)	19.0 (3.0–37.0)	**0.025**	0.4
	No	6.0 (0.0–19.5)	4.0 (1.0–21.0)	6.0 (0.8–21.2)		
Second-line analgesic treatment	Yes	22.0 (9.0–41.0)	25.0 (11.0–40.0)	30.0 (14.0–41.0)	**<0.001**	**0.018**
	No	4.0 (0.8–19.2)	5.0 (1.0–23.2)	6.0 (1.0–22.0)		
Baseline CHFS≥PASS	Yes	41.0 (36.0–48.2)	40.0 (30.0–49.2)	39 (31.0–49.0)	**<0.001**	**0.007**
	No	4.0 (1.0–13.0)	7 (1.0–20.0)	6 (1.0–20.0)		

Bold values denote statistical significance at the p < 0.05 level.

ACA, anti-centromere antibody; CHFS, Cochin Hand Function Scale; mRSS, modified Rodnan Skin Score; PASS, patient acceptable symptom state; RCS, Raynaud’s Condition Score; VAS, visual analogue scale.

### Trajectories and timepoint interaction of CHFS changes

**Figure 2:**
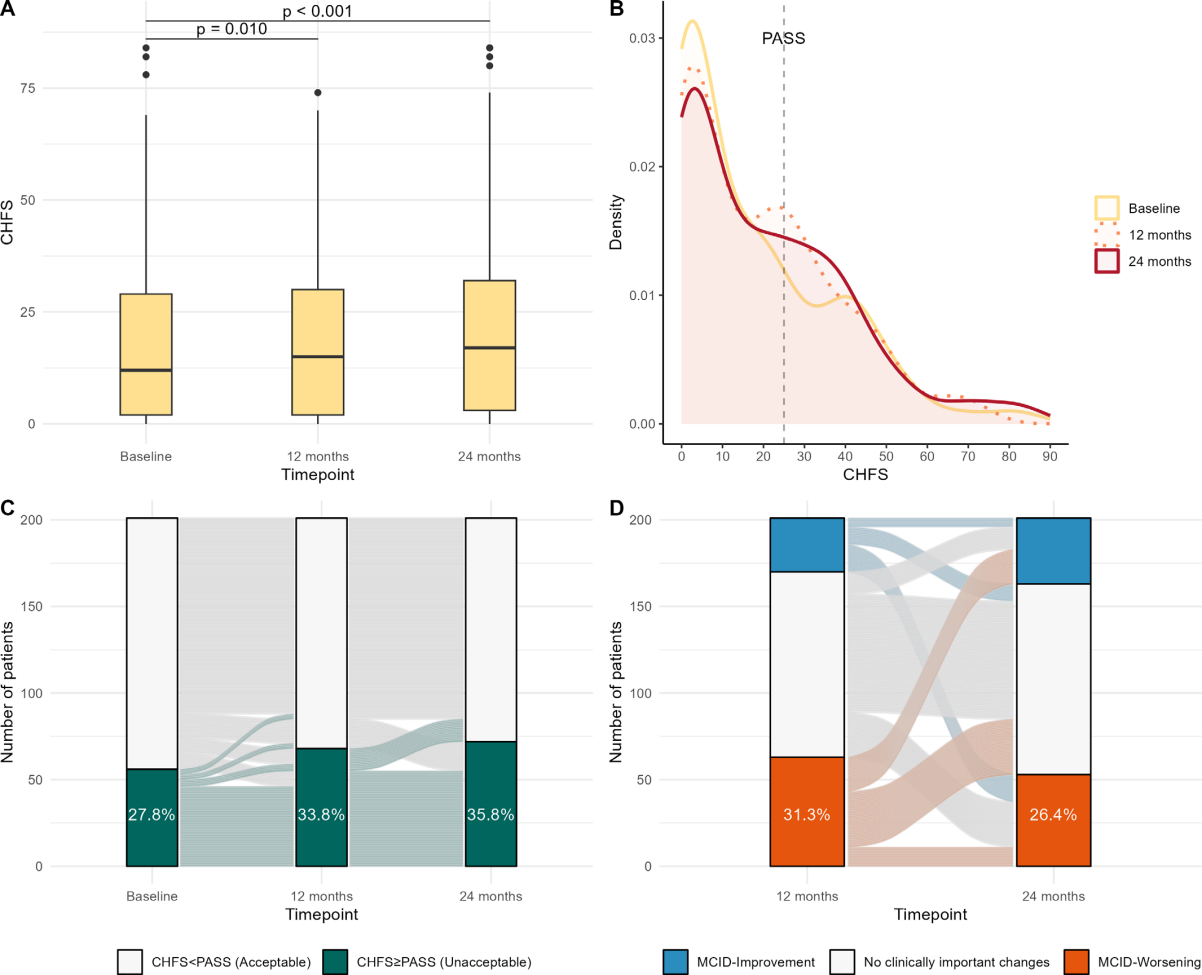
Clinical trajectories in hand function. (A) Density plot showing CHFS distribution changes across the timepoints, (B) alluvial plot showing proportion of patients with CHFS≥PASS and single-patient clinical trajectories across the timepoints, (C) alluvial plot showing proportion of patients with MCID after 12 and 24 months. CHFS, Cochin Hand Function Scale; MCID, minimal clinically important difference; PASS, patient acceptable symptom severity.

Twenty-four months CHFS was available for all patients according to the inclusion criteria for analysis. Twelve months CHFS was missing in recorded in 174 patients. Twenty-seven missing values were imputed as described in methods and shown in online supplemental figure 2. The CHFS assessment over the 24-month follow-up revealed that, despite the reported standard of care, hand disability presented a significant worsening over time in the entire cohort (p<0.001). Specifically, median (IQR) CHFS increased from 12.0 (2.0–29.0) at baseline to 15.0 (IQR 2.0–30.0) at 12 months (p=0.010) and to 17.0 (IQR 3.0–32.0) at 24 months (p<0.001) (figure 2A). The CHFS distribution comparison at the three timepoints revealed a progressive smoothing in the density peak at lower CHFS values and a parallel increase in the number of patients reporting CHFS values close to the 26-point cut-off of PASS (figure 2B). Accordingly, the number of patients reporting CHFS≥PASS increased from 56 (27.9%) at baseline to 68 (33.8%) at 12 months (p=0.025) and 72 (35.8%) at 24 months (p=0.006) (figure 2C).

Interestingly, the alluvial plot also shows that only 6 of 56 patients (10.7%) moved from unacceptable to acceptable CHFS from baseline to 12 months and 13 of 68 patients (19.1%) from 12 to 24 months. The corollary of this observation is that unacceptable hand function was irreversible for more than 80% of patients in the 24-month observation.

Shifting the analysis to MCID in CHFS (figure 2D), by the end of the follow-up, a total of 105 patients (52.2%) experienced MCID-Worsening in at least one of the two 12-month intervals. Specifically, 52 patients (25.9%) experienced the MCID-Worsening only in the first 12-month interval, 42 (20.9%) only in the second and 11 (5.5%) in both. Of note, 20 patients of the 52 with MCID-Worsening in the first year, reported an MCID-Improvement in the following 12-month interval. Nevertheless, since MCID-Worsening is associated with larger absolute and relative CHFS change cut-offs compared with MCID-Improvement, only 10 patients moved back at the end of follow-up to a CHFS equal or inferior to baseline. Hence, also MCID analysis suggested that hand function rarely improves with current standard of care, and it tends to be progressively worse over time.

In the subgroup of 60 patients with the diffuse cutaneous variant, 23 (38.3%) and 17 (28.3%) experienced a CHFS MCID-Worsening between the baseline and the 12-month follow-up, and between the 12-month and the 24-month follow-up, respectively. Furthermore, 11 (18.3%) and 4 (6.7%) of the sample experienced a clinically meaningful progression of skin fibrosis in the corresponding intervals, which was not associated with CHFS MCID-Worsening (p=0.846 and p=0.117, respectively). In the subgroup of 66 patients with ILD, 22 (33.3%) and 19 (28.8%) experienced a CHFS MCID-Worsening from baseline to the 12-month follow-up, and between the 12-month and the 24-month follow-up, respectively. Additionally, 7 (10.6%) and 11 (16.7%) of the sample experienced a PFT deterioration according to PFT in the corresponding intervals, which was not associated with CHFS MCID-Worsening (p=0.120 and p=0.808, respectively).

**Figure 3:**
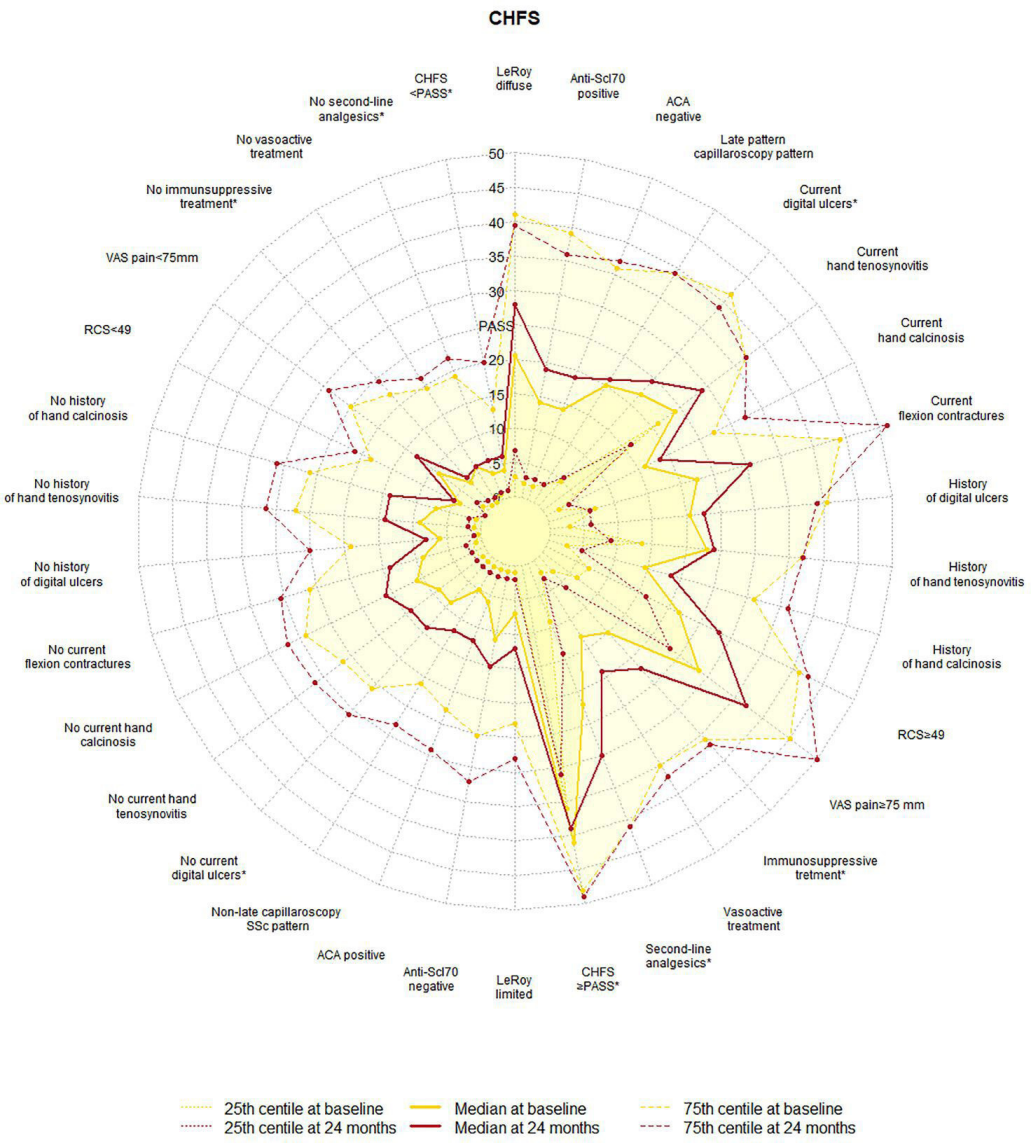
Kiviat chart comparisons of CHFS at baseline and after 24 months in specular clinical variables. *p<0.05 for interaction between the clinical variable and the timepoints at the two-way mixed ANOVA. ACA, anti-centromere antibody; ANOVA, analysis of variance; CHFS, Cochin Hand Function Scale; HAQ-DI, Health Assessment Questionnaire disability index; mRSS, modified Rodnan Skin Score; PASS, patient acceptable symptom state; RCS, Raynaud’s Condition Score; VAS, visual analogue scale.

A comparative two-dimensional visualisation of CHFS increase at 24 months according to the collected categorical variables is shown in figure 3. The radar plot clearly shows the difference in CHFS worsening at 24 months in subgroups of patients defined according to the main categorical clinical variables. The absence of digital ulcers at baseline, the exposure to immunosuppressant or to second-line analgesics, and a baseline CHFS<PASS were the conditions statistically associated with a wider deterioration in hand function over time (table 2). The graphical comparison of baseline and intermediate 12-month timepoint is plotted in online supplemental figure 3.

While only exposure to immunosuppressants or second-line analgesics, RP severity, pain severity and global disability severity statistically differ between patients who met MCID-Worsening criteria within 24 months at the univariate analysis (online supplemental table 1), 13 clinical variables were selected by LASSO analysis of factors associated with MCID-Worsening. Specifically, the variables positively associated with a higher probability of MCID- Worsening were male gender, LeRoy diffuse variant, shorter disease duration, current hand tenosynovitis, higher RCS, higher VAS pain scores, and higher baseline disability according to HAQ-DI. A MCID-Worsening was also more likely in patients exposed to immunosuppressants, vasoactive medications or second-line analgesic treatment. Current digital ulcers, late capillaroscopy scleroderma pattern and higher baseline hand impairment according to CHFS were conversely associated with reduced chances of MCID-Worsening (table 3).

**Table 3:** LASSO regression model for MCID-Worsening

Baseline clinical variables	LASSO regression coefficients
Age, years	–
Male gender	0.211
Le Roy limited variant	−0.430
Disease duration, years	−0.015
ACA positive	–
Anti-Scl70 antibody positive	–
Late SSc capillaroscopy pattern	−0.118
Current digital ulcers	−0.076
Current hand calcinosis	–
Current hand tenosynovitis	0.467
Current flexion contractures	–
Skin score up to elbows	–
VAS pain, mm	0.003
RCS	0.012
HAQ-DI	0.845
Immunosuppressive treatment	0.473
Vasoactive treatment	0.081
Second-line analgesic treatment	0.519
CHFS	−0.054
Intercept	−0.704

ACA, anti-centromere antibody; CHFS, Cochin Hand Function Scale; HAQ-DI, Health Assessment Questionnaire disability index; LASSO, least absolute shrinkage and selection operator; MCID, minimal clinically important difference; RCS, Raynaud’s Condition Score; VAS, visual analogue scale.

Finally, there were 22 deaths reported during the follow-up period, and 9 of these were related to SSc. A baseline CHFS≥PASS was not associated with SSc-related mortality (sHR 1.52, 95% CI 0.64 to 3.60, p=0.340). Similarly, the occurrence of CHFS MCID-Worsening in any of the two 12-month intervals was not associated with SSc-related mortality (sHR 0.62, 95% CI 0.27 to 1.44, p=0.270).

## Discussion

Despite hands are primarily affected in scleroderma, they drive diagnosis and classification of disease and they have been considered a primary driver of disability, hand function is poorly addressed in clinical trials. Our study aimed to describe the natural history of hand function changes over a 24-month interval in a multicentre prevalent SSc cohort. Both differences in CHFS values and clinically meaningful changes, according to previously proposed cut-offs were considered. Further, both the role of single variables and their interactions in multivariable models were addressed.

The baseline assessment of hand function revealed that at least one SSc patient over four reported an unacceptable severity of hand symptoms according to PASS cut-off in a cohort with a median disease duration of 5 years. This symptomatic hand involvement showed, therefore, a prevalence that is comparable to symptomatic pulmonary or cardiac disease in SSc.^32^ The cross-sectional comparisons of hand function revealed an asymmetrical distribution of hand disability burden at baseline according to the single main clinical variables. Patients with diffuse cutaneous variant, history of digital ulcers and advanced capillaroscopy findings, severe RP experience, and current flexion contractures or tenosynovitis reported the worst hand function as partially suggested by previous studies.^4–7^ Unsurprisingly, patients with higher degrees of disability were more likely to be exposed to immunosuppressants and vasoactive medication as well as to second-line treatment for pain control, highlighting the role of pain experience on functional impairment.

One of the most interesting findings of our analysis is that approximately half of the patients reported an MCID-Worsening over 2 years and the total percentage of patients with CHFS≥PASS increased steadily over time. Of note, the proportions of patients who reported an MCID-Worsening and MCID-Improvement were similar at 12-month and 24-month timepoints suggesting that the prevalent nature of the cohort did not constitute an important bias in the analysis.

Further, our analysis also indicated that worsening mainly involved patients with lower CHFS at the beginning of the observation and that only 7 out of 56 patients (12.5%) moved back from an unacceptable to acceptable symptom severity in the 24-month follow-up. Therefore, our longitudinal analysis suggested that hand disability emerged as a progressive process that is cumulative over time and largely irreversible, at least within 24-month observation and with the current standard of care treatments.

Only few variables, namely baseline HAQ-DI, RCS, VAS pain, and exposure to immunosuppressants and second-line analgesics, were associated with an MCDI-Worsening over time. This observation underpinned a difficulty in predicting the short-term evolution of hand disability over time from single clinical variable similar to other organ-specific complications of the disease such as lung or skin. This observation is not surprising, given the specific multifactorial origin of hand functional impairment that could be ascribed to vascular, inflammatory or fibrotic mechanisms of SSc. The LASSO approach to multivariate logistic regression analysis identified an informative set of variables that should be considered in the design of intervention studies targeting hand function in SSc.

Beyond the considerations for target population of intervention trials. the variables identified by the LASSO regression provided interesting insights into putative mechanisms of hand disability SSc. In particular, the negative relationship of MCID-Worsening and disease duration suggested that it could be predominant in the early stages of the disease and that risk factor for severe visceral involvement, such as male gender and diffuse cutaneous disease, are also risk factors for hand function deterioration.

The vascular manifestations of the disease clearly played a leading role in hand function deterioration since RP severity according to RCS was a predictor of functional worsening possibly at the early stages. The observation that the absence of digital ulcers, and late capillaroscopy pattern were indeed all associated with the absence of a functional worsening suggest that the microvascular complications seemed ‘end stage’ features associated with worse function but not causing further worsening in hand impairment. Of note, the specific information provided by the capillaroscopy assessment, highlighted the important role of this tool in the risk-stratification of SSc patients.

The observation of a positive association between active tenosynovitis and MCID-Worsening could indicate a possible time-dependent progressive articular damage in patients with a tenosynovitis-associated SSc, similar to what has been observed in rheumatoid arthritis.^33^

Finally, although the patients with the more severe hand function were more actively treated, immunosuppressants, vasoactive medications and second-line analgesics still showed a positive association with MCID-Worsening when all the other meaningful predictors are considered in the regression models. In our opinion, this observation indeed suggest the insufficiency of these interventions in preventing hand function deterioration in SSc and identify hand function as a clinically unmet need in SSc.

Our study carries some limitations that must be considered in the interpretation of data. The prevalent, observational nature of the cohort is a main factor that limits the interpretation of incident hand disability in SSc. Importantly, hand disability was evaluated through a patient-reported outcome and we lacked an objective measure of tenosynovitis, or a physician recorded clinical measure of overall hand function. Further, digital ulcers, calcinosis and musculoskeletal involvement were not measured on a quantitative scale but recorded as categorical, historical variables. The CHFS cut-offs adopted as part of the analysis, despite being proposed from the analysis of a randomised controlled trial cohort of SSc patients have not been fully validated in an independent population. Finally, non-pharmacological interventions such as hand rehabilitation programmes were not included in the analysis. However, none of the patients included in the study were enrolled in formal, repeated rehabilitation programmes and short-term physiotherapy treatments are known to lose their benefits shortly after discontinuation.^34 35^

## Conclusion

Hand function deteriorations in SSc emerged as a clinically unmet need, with a progressive and mostly irreversible nature despite the currently available treatments. Half of the patients showed a clinically meaningful functional deterioration over 2 years that lead to an irreversible unacceptable symptom state in more than 80% of cases, at least within the 24-month available follow-up. Hand function worsening is multifactorial and transversally involves patients with different disease subsets, autoantibody specificity and clinical phenotype. The use of multivariate adjusted model is therefore the best risk-stratification strategy. Hand function could be considered as outcome measure for future trials in SSc and the reported rate and clinical association with deterioration should be considered in their design.

## Data Availability

Data are available upon reasonable request. The authors confirm that the data supporting the findings of this study are available within the article or its supplementary materials.
