# Supplementary material for: Clinical trajectories of hand function impairment in systemic sclerosis: an unmet clinical need across disease subsets
Source: RMD Open. 2024 Jan 12;10(1):e003216. doi: 10.1136/rmdopen-2023-003216 (PMC10806527; doi:10.1136/rmdopen-2023-003216)
Supplement: Supplementary data [file rmdopen-2023-003216supp001.pdf]

Supplementary Table 1: Univariable association with MCID-Worsening within 24 months

	No MCID-Worsening N = 96	MICID-Worsening N = 105	p-value
**Age**, years, mean±SD	56.0±12.5	55.4±12.0	0.6
**Gender**			0.2
Female	86 (89.6%)	88 (83.8%)	
Male	10 (10.4%)	17 (16.2%)	
**Le Roy variant**			0.2
Diffuse cutaneous	24 (25.0%)	36 (34.3%)	
Limited cutaneous	72 (75.0%)	69 (65.7%)	
**Disease duration**, years, median (IQR)	5.0 (2.0, 13.0)	5.0 (1.0, 9.0)	0.2
**ACA positive**	44 (45.8%)	46 (43.8%)	0.8
**Anti-Scl70 antibody positive**	26 (27.1%)	27 (25.7%)	0.8
**Late capillaroscopy pattern**	47 (49.0%)	41 (39.0%)	0.2
**mRSS**, median (IQR)	2.0 (0.0, 5.0)	2.0 (0.0, 5.0)	>0.9
**Skin score up to elbows**, median (IQR)	2.0 (0.0, 4.0)	2.0 (0.0, 4.0)	0.8
**Current digital ulcers**	17 (17.7%)	11 (10.5%)	0.14
**Any history of digital ulcers**	47 (49.0%)	51 (48.6%)	>0.9
**Current hand calcinosis**	15 (15.6%)	13 (12.4%)	0.5
**Any history of hand calcinosis**	36 (37.5%)	40 (38.1%)	>0.9
**Current hand tenosynovitis**	5 (5.2%)	12 (11.4%)	0.11
**Any history of hand tenosynovitis**	7 (7.3%)	15 (14.3%)	0.11
**Current flexion contractures**	10 (10.4%)	12 (11.4%)	0.8
**VAS pain**, mm, median (IQR)	20.0 (5.0, 60.0)	45.0 (20.0, 65.0)	**0.007**
**VAS pain ≥75 mm**	9 (9.4%)	15 (14.3%)	0.3
**RCS**, median (IQR)	20.0 (5.0, 60.0)	50.0 (20.0, 70.0)	**<0.001**
**RCS ≥49**	32 (33.3%)	58 (55.2%)	**0.002**
**HAQ-DI**, median (IQR)	0.8 (0.0, 1.8)	1.3 (0.9, 1.9)	**0.002**
**Baseline CHFS**	6.5 (0.0, 39.0)	13.0 (3.0, 24.0)	0.3
**Baseline CHFS≥PASS**			0.10
Yes	64 (66.7%)	81 (77.1%)	
No	32 (33.3%)	24 (22.9%)	
**Immunosuppressive treatment**	45 (46.9%)	74 (70.5%)	**<0.001**
**Vasoactive treatment**	73 (76.0%)	88 (83.8%)	0.2
**Second-line analgesic treatment**	33 (34.4%)	52 (49.5%)	**0.030**

Abbreviations: ACA anti-centromere antibody, CHFS Cochin hand function scale, HAQ-DI Health Assessment Questionnaire disability index, IQR interquartile range, mRSS modified Rodnan skin score, PASS patient acceptable symptom state, RCS Raynaud's condition score, SD standard deviation, VAS visual analogue scale.

Supplementary Figure 1: QQ plots of continue variables involved in the analysis

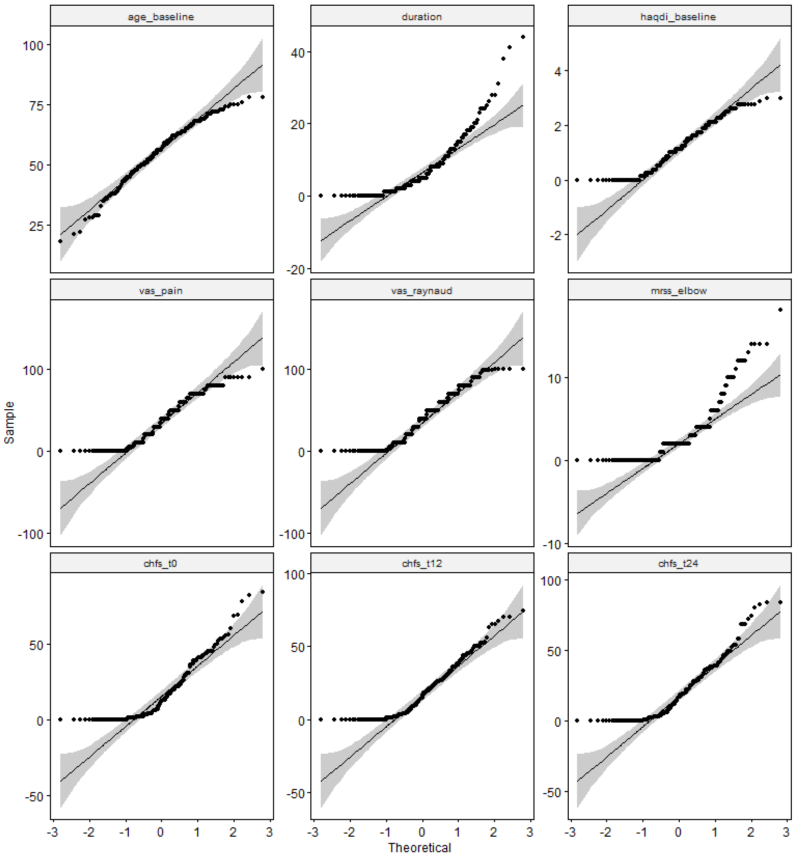

Abbreviations: CHFS Cochin hand function scale, HAQ-DI Health Assessment Questionnaire disability index mRSS modified Rodnan skin score, QQ quantile-quantile, VAS visual analogue scale.

Supplementary Figure 2: Density plots comparing the distribution of the observed CHFS values at 12 months and the five imputed datasets of the missing values

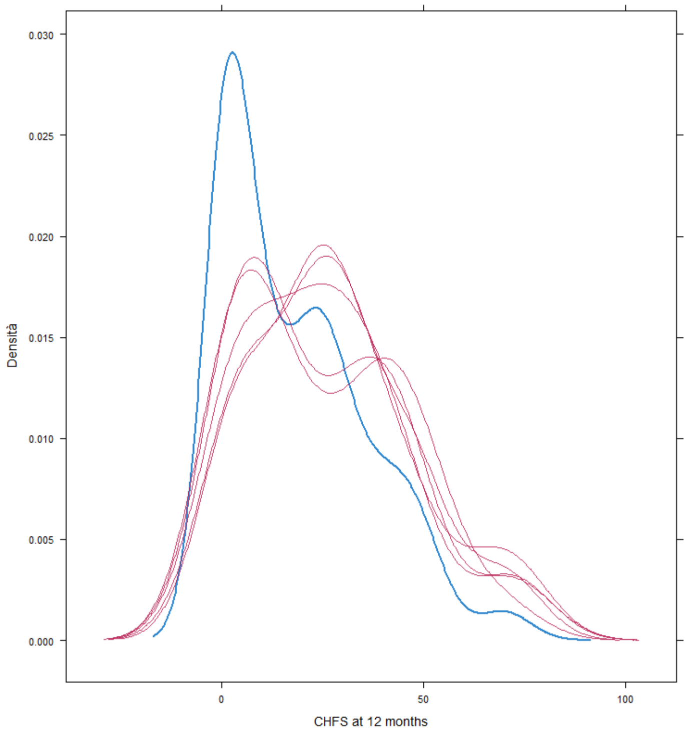

Abbreviations: CHFS Cochin hand function scale

Supplementary Figure 3: Kiviat chart comparisons of CHFS at baseline and after 12 months in specular clinical subsets

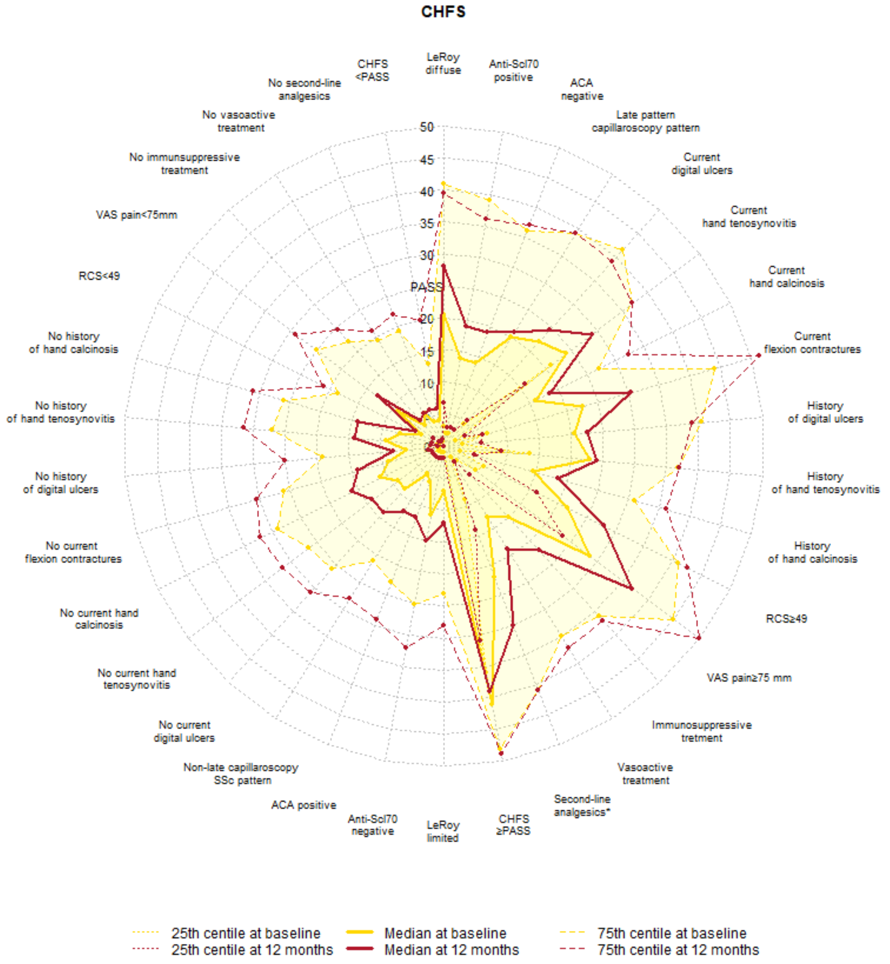

Abbreviations: ACA anti-centromere antibody, CHFS Cochin hand function scale, HAQ-DI Health Assessment Questionnaire disability index, mRSS modified Rodnan skin score, PASS patient acceptable symptom state, RCS Raynaud's condition score, VAS visual analogue scale.
